# Primary Oral Malignant Melanoma: Two Case Reports and Review of Literature

**DOI:** 10.1155/2012/975358

**Published:** 2012-07-30

**Authors:** Neeraj Sharma

**Affiliations:** Department of Oral Medicine and Radiology, Dr. Hsj Institute of Dental Sciences, Panjab University, Chandigarh 160014, India

## Abstract

Primary malignant melanoma of the oral cavity is a rare neoplasm. The tumors tend to metastasize or locally invade tissue more readily than other malignant tumors in the oral region. The survival of patients with mucosal melanomas is less than for those with cutaneous melanomas. Tumor size and metastases are related to the prognosis of the disease. Early detection, therefore, is important.

## 1. Introduction

Malignant melanoma is a potentially aggressive tumor of melanocytic origin [1]. About 1–8% of all melanomas arise in the oral mucosa [2] and these account for 0.5% of all oral malignancies [3–5]. The most frequently affected oral sites are the palate and the maxillary gingiva. The age of reported patients ranges from 20 to 80 years [1, 5]. The neoplasm is more common in Japan and Africa than in Western countries [5].

The etiopathogenesis of mucosal melanomas is poorly understood; however, it is well documented that the melanocytes migrate to both endodermally derived and ectodermally derived mucosa. The function of these melanocytes in the mucosa is not understood. Like their cutaneous counterparts, oral melanomas (OMs) are believed to arise either from nevus, preexisting pigmented areas, Hutchinson's premalignant lentigo or denovo (30% cases) [6]. 

## 2. Case Reports

### 2.1. Case ****1

A dark 60-year-old male patient visited the Department of Oral Medicine and Radiology with a chief complain of recurrent growth in the lower front region since 20 days. The patient gave a history of excision of similar growth about eight months back. The treatment details and the histopathology reports were not available. The patient gave a history of smoking and alcohol use. Medical history was noncontributory.

**Figure 1:**
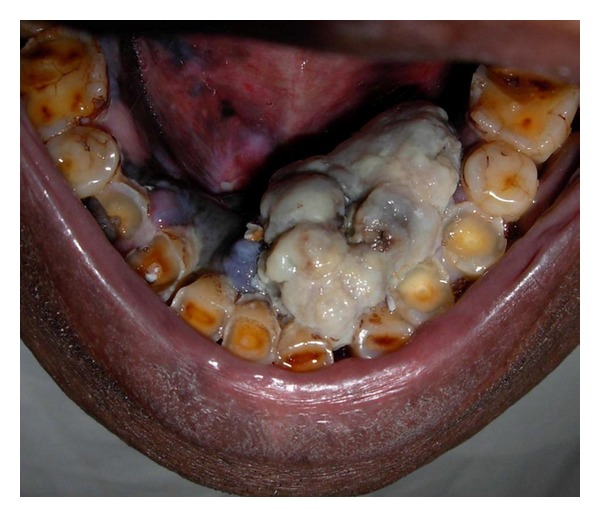
Exophytic growth seen covered with a white pseudomembrane.

**Figure 2:**
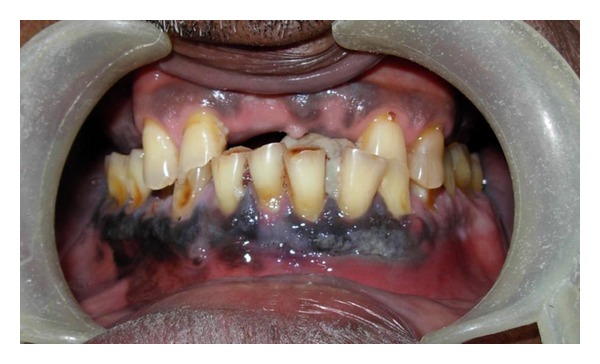
Blackish discoloration of the entire mandibular gingiva.

Extra-oral examination revealed no apparent lymph node enlargement. Intraoral examination showed an exophytic growth of 2.5 × 4 cm approximately on the lingual aspect of mandibular anterior region, extending from the mandibular right lateral incisor upto the left second premolar. The growth was pedunculated, arising from the marginal and attached gingiva of mandibular left incisors and canine. It is well defined, firm, nontender and covered with white pseudomembrane (Figure 1). Removal of the pseudomembrane revealed blackish underlying surface. Mandibular marginal and attached gingiva was black in color especially on the labial/buccal aspect (Figure 2). Multiple satellite lesions could be seen on the floor of the mouth. Correlating all the clinical features a diagnosis of malignant melanoma was made.

**Figure 3:**
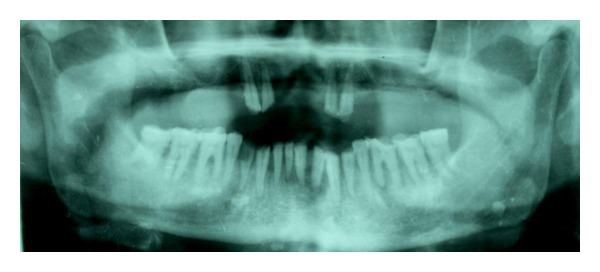
Orthopantomogram showing horizontal bone loss in relation to mandibular incisors and rarefaction in the surrounding bone.

Orthopantomogram showed horizontal bone loss and loss of lamina dura in relation to mandibular incisors. There was generalized rarefaction in the adjoining bone altering the normal trabecular pattern (Figure 3).

**Figure 4:**
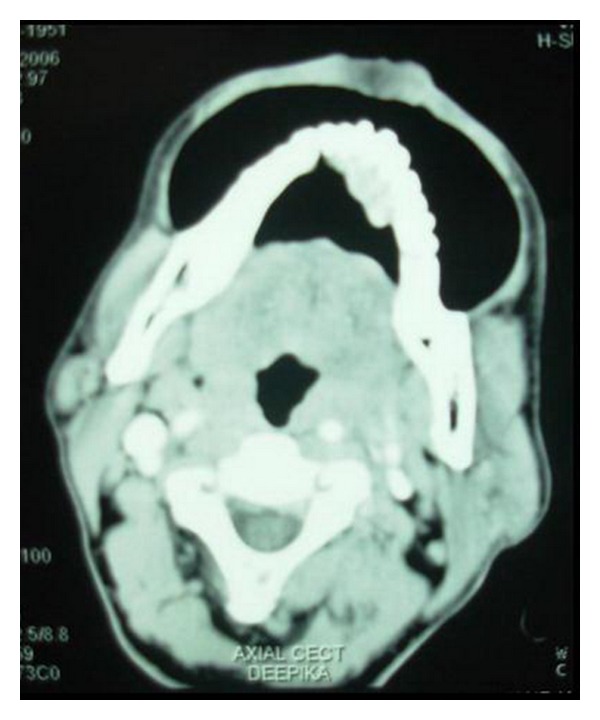
Blow mouth axial CT scan showing enhanced growth.

**Figure 5:**
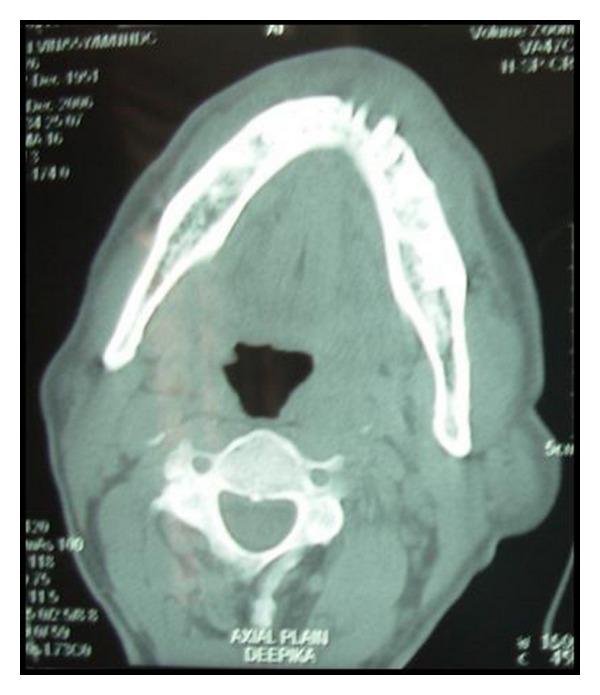
Erosion of the anterior cortex of the left body of mandible seen on the axial CT scan bone window.

Computed tomogram (CT) showed 2.3 × 4.4 cm sized enhancing lesion extending from right mandibular lateral incisor upto the left second premolar lingually (Figure 4). There was erosion of the anterior cortex of the left body of mandible to the symphyseal region (Figure 5). Enlarged, enhancing bilateral level I and II lymph nodes were seen.

### 2.2. Case ****2

**Figure 6:**
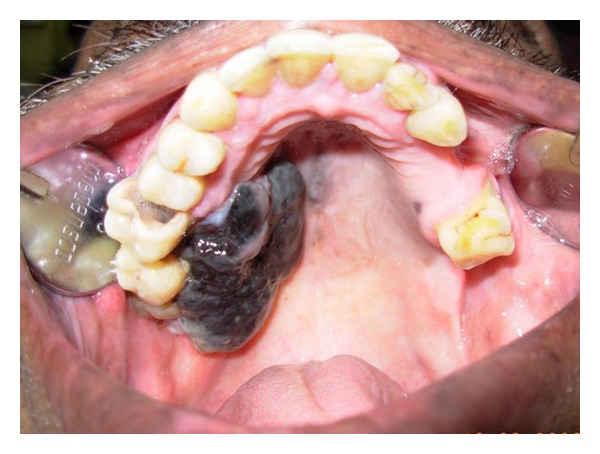
A black exophytic growth was seen on the right side of the hard palate with satellite lesions.

**Figure 7:**
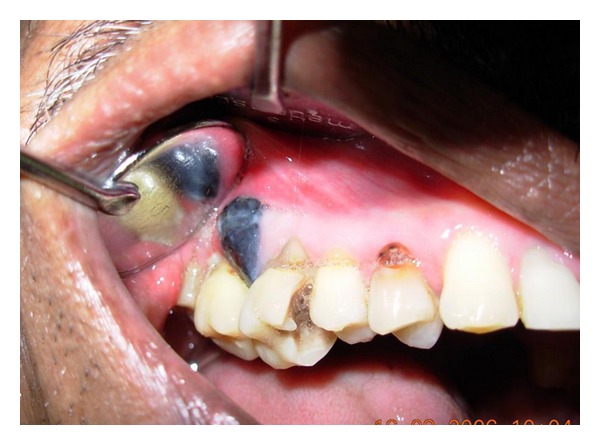
Buccal extension of the lesion involving the gingiva between the maxillary right first and second molars.

A 50-year-old male patient with pain in the maxillary right first molar and a blackish growth on the right side of hard palate since 12 days visited the Department of Oral Medicine and Radiology. The patient did not report taking medications for any other illnesses. Habitual history was negative. Extra-oral examination revealed enlargement of bilateral submandibular lymph nodes, which were oval in shape, mobile, and nontender. On intraoral examination, a black exophytic growth was seen on the right side of the hard palate (Figure 6). The lesion extended onto the buccal aspect involving the gingiva between the maxillary right first and second molars (Figure 7). It was soft in consistency and nontender. Small satellite lesions were seen on the palate surrounding the lesion. A clinical diagnosis of malignant melanoma was made. Radiographs were taken and incisional biopsy performed.

**Figure 8:**
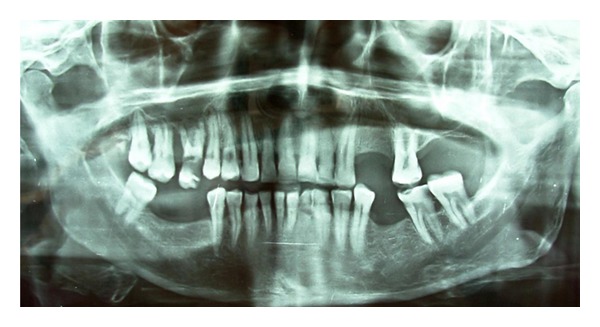
Orthopantomogram showing destruction of interdental bone between maxillary right first and second molars, disruption of the corticated border of the floor of right maxillary sinus.

**Figure 9:**
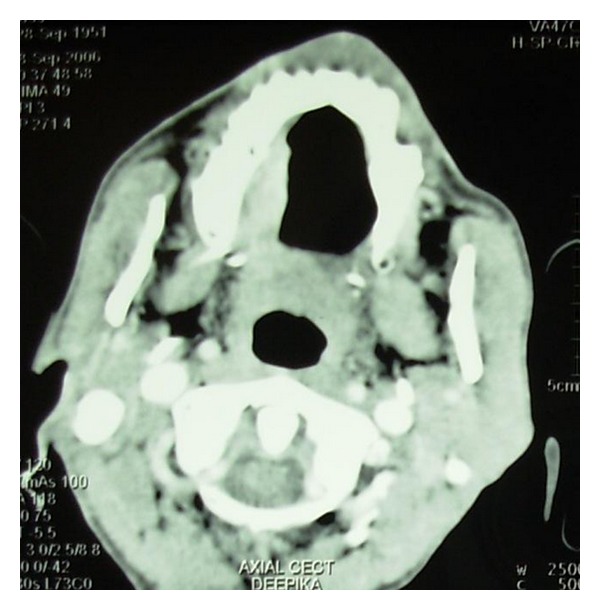
Axial CT scan showing ill-defined enhancing soft tissue mass on right side of hard palate extending posteriorly upto the soft palate.

**Figure 10:**
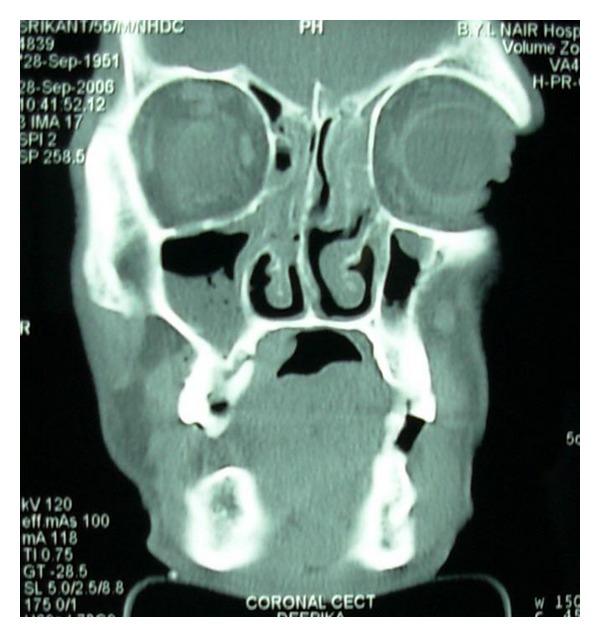
Coronal CT scan at the level of the maxillary right first molar showing bone destruction around the tooth.

Orthopantomogram showed carious maxillary right first and second molars with destruction of the interdental bone between them. The continuity of the floor of the maxillary sinus was disrupted in the maxillary right first molar region. The periodontal ligament space in relation to all the right maxillary molars and second premolar was widened (Figure 8). CT scan showed an ill-defined enhancing soft tissue mass on right side of hard palate, extending posteriorly upto the soft palate. There was no evidence of erosion or sclerosis of bone due to the soft tissue mass (Figures 9 and 10). Bilaterally enlarged level I, II, V lymph nodes were seen.

**Figure 11:**
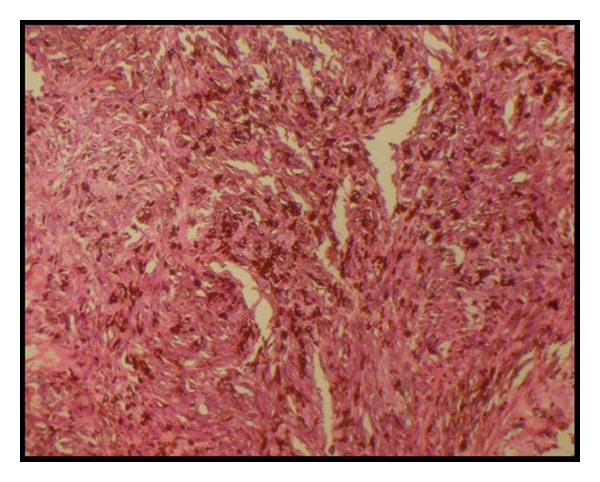
Histopathology slide showing spindle-shaped melanocytes.

Incisional biopsies were taken from both the lesions. Histopathological studies revealed numerous atypical melanocytes within the epithelium and also invasion into the connective tissue. These cells were epitheloid to spindle in shape, with vesicular, hyperchromatic nuclei, and prominent nucleoli. Few mitotic figures were also seen (Figure 11). Thus, the pathological examination confirmed the diagnosis of malignant melanoma. The patients were then referred to the Regional Cancer Institute for further treatment. Both patients underwent excision of the primary lesion along with radical neck dissection and postoperative radiotherapy.

## 3. Discussion

The initial symptom and sign of OM is often a pigmented growth or swelling. The surface may be smooth, with an intact or ulcerated overlying mucosa. Satellite foci may surround the primary tumor. The color may be uniformly brown or black or may show variation of color, with black, brown, grey, purple, and red shades, or depigmentations [7]. In amelanotic melanomas, pigmentation is absent [8]. OM has an initial phase characterized by radial growth followed by a phase of invasion of the underlying tissues (the so-called “vertical growth phase”).

Other presenting signs and symptoms include bleeding, ill-fitting dentures, pain, increased mobility of teeth, and delayed healing of extraction sockets. The OM is more aggressive and the abundant blood supply of the oral cavity may permit blood vessel invasion and hematogenous dissemination early in the course of the disease [2]. Regional lymphadenopathy may be present and connotes a poor prognosis [9]. Clinically, oral melanomas are classified into five types: pigmented nodular, nonpigmented nodular, pigmented macular, pigmented mixed, and nonpigmented mixed [10].

When an oral pigmentation cannot be confidently diagnosed as benign on clinical grounds, a biopsy is mandatory. An excisional biopsy with a 1 to 2 mm margin for small lesions or an incisional biopsy through the thickest or the most suspicious part of the tumor in case of a large lesion is required [11]. Fine needle aspiration or exfoliative cytology of primary pigmented lesions is contraindicated. It has been suggested that cutting into a malignant neoplasm during an incisional biopsy or other invasive procedure could result in accidental dissemination of malignant cells within the adjacent tissues (seeding) or even in the blood or lymphatic stream, with the subsequent risk of local recurrence, or regional or distant metastasis [1]. The most common sites of metastasis are lung, bone, brain, and liver, with widespread involvement occurring in advanced disease [2].

Malignant cells of OM show a wide range of shapes, including spindle, plasmocytoid, clear cell, and epithelioid ones. These malignant cells possess considerable pleomorphism with large, irregular hyperchromatic nuclei, and prominent nucleoli, and have readily detectable mitotic activity. OMs can be histologically subclassified into (1) in situ melanoma, which is limited to the epithelium and the epithelial-connective tissue interface; (2) melanomas with an invasive pattern, in which the neoplasm extends into the connective tissue; (3) melanomas with a combined pattern of invasive melanoma with in situ component [7].

A simple TNM clinical staging, recognizing three stages, has shown to be of prognostic value. A recent histopathological microstaging for Stage I subclassifies it into three levels [1, 12, 13].

 Stage I: Primary tumour present only (Tany N0M0).
   Level I: pure in situ melanoma without evidence of invasion or in situ melanoma with “microinvasion,”
   Level II: invasion up to the lamina propria,
   Level III: deep skeletal tissue invasion into skeletal muscle, bone, or cartilage.
 Stage II: Tumour metastatic to regional lymph nodes (Tany N1M0).
 Stage III: Tumour metastatic to distant sites (Tany Nany M1).

Treatment of OM is still controversial. Excision of the primary lesion, preferably using an intraoral approach and involving at least 1.5 cm of healthy tissue, is recommended [14]. Patients with primary OM present lymph node metastasis in 25% of cases [13]. Neck dissection should be reserved for cases with preoperatively confirmed lymph node metastases and the choice of the neck dissection modality should be guided by the extent and the level of the nodes [8].

Surgery could be combined with radiotherapy, chemotherapy, or immunotherapy even though the effectiveness of such therapies is mostly unknown. Postoperative radiotherapy is generally recommended if poor prognostic pathologic features are present, such as multiple positive nodes, or extranodal spread of metastastic melanoma, even though OMs are regarded as poorly radiosensitive. Other irradiation modalities such as intraoral mould (^60^Co, ^192^Ir, or ^198^Au), intraoral electron beam or interstitial brachytherapy have also been used [15].

Dacarbazine, platinum analogs, nitrosoureas, microtubular toxins, dimethyl triazeno imidazole carboxamide (DTIC), nimustine hydrochloride, or vincristine have been used as adjuvant therapy or postoperative chemotherapy. IFN-*α*2b, IL-2, BCG, anti-Fas antibody, IL2, and cytokines have shown varied results [1].

The prognosis of OM is poor. A tumor thickness greater than 5 mm, presence of vascular invasion, necrosis, polymorphous tumor cell morphology and the inability to properly resect the lesions with negative margins have been associated with poor survival in patients with primary OM [1]. Gingival melanoma has a better 5-year survival rate than palatal melanoma [3]. Recurrences may occur even 10–15 years after primary therapy. Distant metastases to the lungs, brain, liver, and bones are frequently observed [16].

## 4. Conclusion

Primary oral mucosal melanomas are exceedingly rare and biologically aggressive malignancies. OMs clinically mimic many other pigmented lesions of the oral cavity. All oral pigmented lesions that are not clinically diagnostic should be biopsied. Dental and medical practitioners who treat oral lesions should include malignant melanoma in the differential diagnosis of pigmented lesions because early diagnosis and intervention result in better prognosis.
